# Dysregulation of Cortisol Metabolism in Equine Pituitary Pars Intermedia Dysfunction

**DOI:** 10.1210/en.2018-00726

**Published:** 2018-10-04

**Authors:** Ruth A Morgan, John A Keen, Natalie Homer, Mark Nixon, Anna M McKinnon-Garvin, Jodie A Moses-Williams, Sarah R Davis, Patrick W F Hadoke, Brian R Walker

**Affiliations:** 1 University/British Heart Foundation Centre for Cardiovascular Science, The Queen’s Medical Research Institute, University of Edinburgh, Edinburgh, United Kingdom; 2 Royal (Dick) School of Veterinary Studies, University of Edinburgh, Easter Bush Campus, Midlothian, United Kingdom; 3 Institute of Genetic Medicine, Newcastle University, International Centre for Life, Newcastle upon Tyne, United Kingdom

## Abstract

Equine Cushing disease [pituitary pars intermedia dysfunction (PPID)] is a common condition of older horses, but its pathophysiology is complex and poorly understood. In contrast to pituitary-dependent hyperadrenocorticism in other species, PPID is characterized by elevated plasma ACTH but not elevated plasma cortisol. In this study, we address this paradox and the hypothesis that PPID is a syndrome of ACTH excess in which there is dysregulation of peripheral glucocorticoid metabolism and binding. In 14 horses with PPID compared with 15 healthy controls, we show that in plasma, cortisol levels and cortisol binding to corticosteroid binding globulin were not different; in urine, glucocorticoid and androgen metabolites were increased up to fourfold; in liver, 11*β*-hydroxysteroid dehydrogenase type 1 (11*β*-HSD1) expression was reduced; in perirenal adipose tissue, 11*β*-HSD1 and carbonyl reductase 1 expression was increased; and tissue cortisol levels were not measurably different. The combination of normal plasma cortisol with markedly enhanced urinary cortisol metabolite excretion and dysregulated tissue-specific steroid-metabolizing enzymes suggests that cortisol clearance is increased in horses with PPID. We infer that the ACTH excess may be compensatory and pituitary pathology and autonomous secretion may be a secondary rather than primary pathology. It is possible that successful therapy in PPID may be targeted either at lowering ACTH or, paradoxically, at reducing cortisol clearance.

Equine Cushing disease, now more commonly known as pituitary pars intermedia dysfunction (PPID), affects ∼30% of horses >15 years (1). It is characterized by hyperplasia or adenoma formation in the pars intermedia of the pituitary, attributed to loss of inhibitory dopaminergic innervation (2). When first described, the clinical signs of hypertrichosis, insulin dysregulation, muscle wastage, polyuria and polydipsia, and vascular dysfunction (1) were attributed to excess glucocorticoids given their similarity to human and canine Cushing disease. Horses with PPID have elevated plasma ACTH but, paradoxically, do not have elevated plasma cortisol concentrations (3, 4), and as such, the condition is now more commonly termed *PPID* (5). Few attempts have been made to address this paradox; it is suggested that ACTH produced by the diseased pituitary is less biologically active, but limited data support this theory (6, 7) and it does not explain the clinical signs.

Tissue exposure to glucocorticoids is determined by several control mechanisms. The hypothalamic-pituitary-adrenal (HPA) axis determines the secretion of glucocorticoids into the plasma. Once in the plasma, the majority of cortisol is bound to corticosteroid binding globulin (CBG) (8); only unbound cortisol is free to diffuse into the tissues, where it can activate both glucocorticoid receptors (GRs) and mineralocorticoid receptors. In the tissues, access to receptors is further controlled by metabolizing enzymes such as 11*β*-hydroxysteroid dehydrogenase type 1 (11*β*-HSD1) and 11*β*-hydroxysteroid dehydrogenase type 2 (11*β*-HSD2), which interconvert inactive cortisone with active cortisol, thus conferring tissue-specific sensitivity (9). A number of other enzymes such as 5*α*- and 5*β*-reductases (10) and, in the horse, carbonyl reductase 1 (CBR1) (11) metabolize glucocorticoids prior to excretion of metabolites in the urine and feces and may also modulate local receptor activation in tissues where they are active. Dysregulation of any of these control mechanisms alters tissue glucocorticoid exposure, but this is not necessarily reflected in plasma cortisol measurements. For example, in human obesity, increased glucocorticoid clearance results in impaired negative feedback and subtle activation of the HPA axis, thus maintaining normal circulating cortisol concentrations, but tissue cortisol levels may be elevated by increased 11*β*-HSD1 activity (12). Urinary excretion of glucocorticoids is significantly increased in obese horses despite normal plasma cortisol concentrations (11), suggesting a similar compensatory change occurs in this species.

In this study, we address the hypothesis that PPID is characterized by dysregulation of glucocorticoid metabolism and binding, resulting in enhanced tissue glucocorticoid exposure. We further tested whether expression of GRs and cortisol metabolizing enzymes (11*β*-HSD1/2) in the pituitary is altered in PPID.

## Materials and Methods

### Animals

Horses with PPID and healthy controls, all destined for euthanasia, were recruited from clinics at the Royal (Dick) School of Veterinary Studies, with approval from the University of Edinburgh Veterinary Ethical Review Committee. All groups included females and castrated males, reflecting the clinical population in the United Kingdom. The age, breed, sex, body condition score (out of 5) (13), clinical features of previous laminitis, and medical history (specifically history of laminitis and glucocorticoid administration) were recorded. Blood was obtained after overnight fasting, between 9:00 am and 10:00 am, via an intravenous cannula inserted in the jugular vein for the purpose of euthanasia. ACTH and insulin concentrations were measured by chemiluminescent immunoassays (Immulite 2000; Siemens, Camberley, UK), and plasma *α*–melanocyte stimulating hormone [*α*-MSH, a proopiomelanocortin-derived peptide released from the pars intermedia and increased in PPID (14)] was measured by radioimmunoassay (Euria *α*-MSH RIA Kit; Eurodiagnostica, Malmo, Sweden). Horses were humanely euthanized (9:00 am to 10:00 am) with quinalbarbitone sodium and cinchocaine hydrochloride (1 mL/10 kg bodyweight) (Somulose; Dechra Veterinary Products, Shrewsbury, UK). Samples of neck crest adipose, perirenal adipose, linea alba adipose, and liver were snap frozen and stored at −80°C. Urine was collected at postmortem (9:00 am to 10:00 am). The horses had free access to water overnight. Preliminary work demonstrated a diurnal rhythm to urinary metabolite excretion with the peak at midday; samples were collected just prior to this peak in excretion. The pituitaries were harvested, bisected, and fixed in 10% formalin for histological examination and snap frozen for quantitative PCR.

Healthy horses were defined as those with no clinical, histological, or biochemical evidence of endocrine disease and no history of glucocorticoid administration within the previous 3 months. Horses with PPID were defined as those older than 15 years with one or more clinical sign of PPID (hypertrichosis, supraorbital fat pads, laminitis, history of laminitis, polyuria, and polydipsia), elevated plasma ACTH (>100 pg/mL), and/or elevated *α*-MSH (>90 pmol/L) (14) and histological evidence of hyperplasia, microadenoma, or adenoma of the pars intermedia (grade ≥3/5) (15).

### Quantification of glucocorticoids in plasma, adipose, and liver tissue

Glucocorticoids [cortisol, cortisone, corticosterone, 11-dehydrocorticosterone, and 20*β*-dihydrocortisol (20*β*-DHF)] were extracted from plasma (200 μL) (16), liver (100 mg), and adipose (100 mg); separated; and quantified by liquid chromatography–tandem mass spectrometry (LC-MS/MS) as previously described (11).

### Quantification of androgens in plasma

Plasma androgens (testosterone and androstenedione) were analyzed by adapting a previously described method (17). Briefly, steroids were extracted from plasma (500 μL) by solid-phase extraction on an HLB Oasis (60-mg, 3-cc columns; Waters UK, Elstree, UK) with 10 ng ^13^C_3_-testosterone and ^13^C_3_-androstenedione (Sigma Aldrich, Dorset, UK) as internal standards. Extracted steroids were separated using liquid chromatography on a UPLC column (2.1 mm × 50 mm, 1.7 μm; Acquity UPLC BEH C18; Waters, MA). All steroids were analyzed in positive ion mode using electrospray ionization on an AB Sciex QTrap 5500 operating in triple quadrupole mode for testosterone (*m/z* 289 → 97, 25 V) and androstenedione (*m/z* 287 → 97, 25 V). Linear regression analysis of calibration standards, calculated using peak area ratios of analytes to internal standard, was used to determine the concentration of the analytes in the samples.

### Urinary steroid analysis by mass spectrometry

Free and conjugated steroids were extracted from urine (20 mL) from a subset of animals (healthy controls, n = 10; PPID, n = 10) by solid-phase extraction on Bond Elut Nexus mixed-mode Large Reservoir Capacity, 60-mg columns (Agilent Technologies, Santa Clara, CA). Briefly, steroid conjugates were hydrolyzed using *β*-glucuronidase followed by reextraction. The steroids obtained were derivatized to form methoxime-trimethylsilyl derivatives. Steroidal derivatives were separated by gas chromatography using a 35HT Phenomenex column (30 m × 0.25 mm × 0.25 μm; Agilent Technologies) on a TRACE GC Ultra Gas Chromatograph (Thermo Fisher Scientific). Mass analysis was performed on a TSQ Quantum Triple Quadrupole tandem mass spectrometer (Thermo Fisher Scientific, Waltham, MA) as previously described (18). Epicortisol and epitetrahydrocortisol were used as internal standards (Steraloids, Newport, RI). The steroids analyzed were cortisol, cortisone, 5*β*-tetrahydrocortisol, 5*β*-tetrahydrocortisone, 5*α*-tetrahydrocortisol, *α*-cortol, *β*-cortol, *α*-cortolone, *β*-cortolone, testosterone, aetiocholanolone, and androsterone, as previously described (18). In addition, 6*β*-hydroxycortisol (*m/z* 693.5 → 513.3) and the isomers 20*α*-dihydrocortisol and 20*β*-DHF (*m/z* 681.4 → 488.3), which had distinct chromatographic separation, were monitored.

Steroid quantities are expressed as a ratio to creatinine, which was measured using a colorimetric method based on the modified Jaffe reaction (IL650 analyzer; Instrumentation Laboratories, Barcelona, Spain). Urinary creatinine concentrations did not differ significantly between the groups (healthy 20.3 ± 2.1 vs PPID 21.4 ± 2.2 mmol/L).

### CBG

CBG binding capacity was measured as previously described (19). Briefly, plasma samples were diluted (1:100), stripped of endogenous steroids using dextran-coated charcoal, and incubated with [1,2,6,7]-^3^H_4_ cortisol (^3^H-cortisol) in the presence and absence of unlabeled cortisol to assess nonspecific binding. Free ^3^H-cortisol was removed by incubation with dextran-coated charcoal, and the remaining CBG bound ^3^H-cortisol quantified by scintillation spectrophotometry. Free cortisol fraction was determined as previously described (20). Briefly, serum samples were diluted (1:5) and incubated with ^3^H-cortisol. Radioactivity was determined, by scintillation spectrophotometry, before and after ultrafiltration. Free cortisol concentrations were then calculated from the total concentration previously measured by LC-MS/MS. Total plasma CBG was measured by ELISA (MyBioSource, San Diego, CA).

### Processing of pituitary tissue

Sections (5 μm) of the frozen embedded tissue were cut and stained with hematoxylin and eosin. The stained section was then examined under a microscope to identify the pars intermedia and pars distalis. Core biopsies of each of these areas were taken for mRNA quantification. Sections (5 μm) from tissue samples fixed for 24 hours in formalin were subject to immunohistochemical analysis for the presence of GRs. Slides were deparaffinized with xylene and hydrated in decreasing concentrations of ethanol (100%, 95%, 70% in distilled water). Endogenous peroxidase activity was blocked with hydrogen peroxide [3% v/v in PBS; 5 minutes, room temperature (RT)]. Nonspecific staining was blocked with the addition of BSA (2.5% v/v in PBS; 1 hour), followed by addition of goat serum (20% v/v in PBS, 30 minutes). Slides were incubated with primary antibody (SC-1003; Santa Cruz Biotechnology, Santa Cruz, CA) (21) (diluted 1/100 in PBS/1% BSA; 30 minutes, RT) and then washed in PBS. Slides were incubated with goat anti-rabbit secondary antibody (Vector Laboratories, Burlingame, CA) (diluted 1/400 in PBS/1% BSA) for 30 minutes at RT and then washed in PBS. Extravidin Peroxidase LSAB reagent diluted 1/200 in PBS/1% BSA was added to the sections and incubated for 30 minutes. The slides were again washed in PBS prior to application of diaminobenzidine tetrahydrochloride (Vector Laboratories) solution.

### mRNA quantitation

Total RNA was extracted from adipose, liver, pars distalis, and pars intermedia using the RNAeasy Mini Kit (Qiagen, Valencia, CA). The tissue was mechanically disrupted in either QIAzol (Qiagen) for adipose tissue or RLT buffer (Qiagen) for liver and pituitary tissue. RNA quality was assessed using a Nanodrop Spectrometer and confirmed by electrophoresis using a 1% agarose gel. Then, 500 ng RNA was reverse transcribed using the Quantitect Reverse Transcription Kit (Invitrogen, Carlsbad, CA) to synthesize cDNA.

Quantitative real-time PCR was performed using a Light-cycler 480 (Roche Applied Science, Indianapolis, IN). Primers were designed using sequences from the National Centre of Biotechnological Information and the Roche Universal Probe Library (Roche Applied Sciences) (22). Target gene expression was arbitrarily quantified against a standard curve constructed from pooled samples for each primer probe combination. Amplification curves were plotted for each sample (y = fluorescence, x = cycle number). Triplicates were deemed acceptable if the standard deviation of the crossing point was <0.4 cycles. The standard curve generated for each gene (y = crossing point, x = log concentration) was deemed acceptable if the reaction efficiency was between 1.7 and 2.1. The abundance of each gene was expressed relative to two housekeeping genes and expressed as arbitrary units. The most appropriate housekeeping genes for each tissue were determined by testing six candidates and using NormFinder software to identify those with least inter- and intrasample variation (23) out of six tested [*β*-actin, hypoxanthine-guanine phosphoribosyltransferase (*HPRT*), succinate dehydrogenase A (*SDHA*), TATA-box binding protein, glyceraldehyde 3-phosphate dehydrogenase, and 18S]. The following housekeeping combinations were used: neck crest adipose, *β*-actin and *SDHA*; perirenal adipose, 18S, and *SDHA*; linea alba adipose, 18S, and *SDHA*; liver, *SDHA*, and *HPRT*; and pituitary, 18S, and *HPRT*.

### Statistical analysis

A power calculation, based on prestudy estimates and previously published data of what would constitute a biologically meaningful difference (11), determined that a sample size of eight per group would give 85% power to detect a 25 ± 15–μg/mmol creatinine difference in total cortisol metabolite excretion (*α* < 0.05). Statistical analysis was performed using GraphPad Prism 5 (GraphPad Software, La Jolla, CA) and SPSS Statistics 19 (IBM Software, New York, NY). Categorical data were analyzed by the Fisher exact test. Continuous data were tested for normality using a Kolmogorov-Smirnov test. Data from the two groups were analyzed using a Student *t* test or Mann-Whitney *U* test. The effect of sex on the various measurements was determined by two-way ANOVA.

## Results

### Clinical characteristics

Horses with PPID were older and had substantially elevated plasma ACTH and *α*-MSH compared with healthy controls (Table 1). Body condition score and serum insulin did not differ between the groups, although insulin tended to be higher in horses with PPID. Pars intermedia histopathology score was higher in horses with PPID, indicating pathology of the pars intermedia (15). Serum albumin (healthy 33.95 ± 0.7 vs PPID 33.04 ± 0.63 g/L), *γ*-Glutamyl transferase and glutamate dehydrogenase were within normal limits and did not differ significantly between the groups. There were fewer females in the healthy group than in the group with PPID, but this difference was not statistically significant.

**Table 1.** Clinical Characteristics of the Study Groups

	Healthy (n = 15)	PPID (n = 14)
Age, y	15.1 ± 4.7	22.2 ± 3.5*^a^*
Sex	3 Females	7 Females
	12 Castrated males	7 Castrated males
Breeds	7 TB or TBX	5 TB or TBx
	2 Percheron	1 Percheron
	2 ISH	1 ISH
	2 Welsh	3 Welsh
	1 Exmoor	1 Trakhener
	1 WB	2 Shetland
Body condition score, /5	2.2 ± 0.7	2.2 ± 1.0
Serum insulin, IU/L	2.5 ± 1.2	8.3 ± 9.2
Plasma *α*-MSH, pmol/L	20.5 ± 15.0	170.7 ± 81.2*^a^*
Plasma ACTH, pg/mL	31.9 ± 13.9	274.4 ± 90.8*^a^*
Pituitary score, /4 (15)	1.0 (1–2)	4 (4–5)*^a^*

Comparisons between groups were by *χ*^2^ for categorical data (sex and breed). Continuous data were tested for normality using a Kolmogorov-Smirnov and comparisons made by Student *t* test or Mann-Whitney *U* tests. Data are mean ± SD (normally distributed) or median (interquartile range).

Abbreviations: ISH, Irish Sport Horse; TB, Thoroughbred; TBX, Thoroughbred cross; WB, Warmblood.

^a^ *P* < 0.05 compared with healthy horses.

### Measurements of adrenal steroids and CBG in plasma

**Figure 1.**
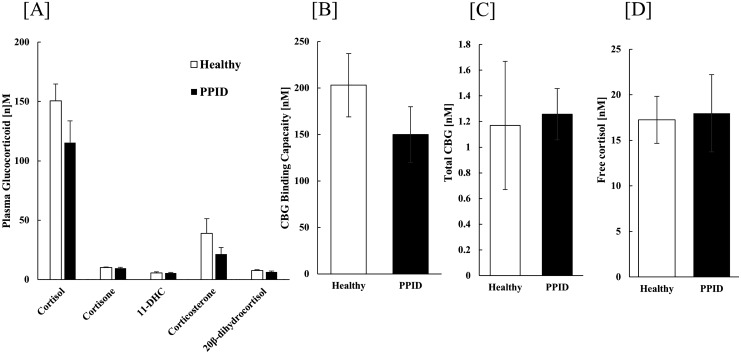
Plasma glucocorticoid concentrations and CBG in healthy horses and horses with PPID. (A) Total plasma cortisol, cortisone, 11-deoxycorticosterone (11-DHC), corticosterone, and 20*β*-DHF concentrations were not significantly different between healthy horses and horses with PPID. (B) CBG binding capacity (nM) for cortisol (C) total plasma CBG content and (D) free cortisol concentrations was not significantly different between the groups. Data are mean ± SEM.

Neither plasma glucocorticoids (cortisol, cortisone, 11-deoxycorticosterone, corticosterone, and 20*β*-DHF) (Fig. 1A) nor total CBG content, CBG binding capacity, free cortisol fraction (percentage of total cortisol; healthy 15.2% ± 6.8% vs PPID 14.0% ± 2.3%), or free cortisol concentration were significantly different in horses with PPID compared with healthy horses (Fig. 1B–1D), although there was a trend for lower binding capacity in PPID (Fig. 1A). Plasma glucocorticoids did not differ between sexes.

Androstenedione was the predominant circulating plasma androgen as previously described (24). Plasma testosterone was significantly lower in castrated males than in females in both groups; plasma androstenedione did not differ with sex (Table 2). Plasma androgens were not different between groups with or without adjustment for sex (Table 2).

**Table 2.** Plasma Androgen Concentrations in Healthy Horses and Horses With PPID

Characteristic	Healthy Female (n = 3)	Healthy Castrated Male (n = 12)	PPID Female (n = 7)	PPID Castrated Male (n = 7)
Testosterone, pg/mL	70.2 ± 15.3	16.3 ± 3.7*^a^*	83.7 ± 15.9	26.2 ± 6.2*^a^*
Androstenedione, pg/mL	89.5 ± 10.4	95.3 ± 23.5	88.7 ± 9.6	72.0 ± 22.6

Data are mean ± SD. Following a Kolmogorov-Smirnov test for normality, comparisons between groups were by Mann-Whitney *U* tests. There were no significant differences between the horses with and without PPID.

^a^ There were significant differences between males and females within (*P* < 0.05) and between each disease group.

### Measurements in urine

Total urinary cortisol metabolite excretion was increased fourfold in horses with PPID compared with healthy horses (Table 3). This was largely accounted for by an increase in 20*β*-DHF excretion (Table 3). Ratios reflecting 5*β*-reduction of cortisol (5*β*-tetrahydrocortisol/cortisol) and cortisone (tetrahydrocortisone/cortisone), 5*α*-reduction of cortisol (5*α*-tetrahydrocortisol/cortisol), renal 11*β*-HSD2 activity (cortisol/cortisone), and overall 11*β*-HSD1 and 11*β*-HSD2 activity (tetrahydrocortisols/tetrahydrocortisone) were not different between the groups.

**Table 3.** Urinary Glucocorticoid Metabolite Excretion in Healthy Horses and Horses With PPID

Metabolite (μg/mmol Creatinine)	Healthy (n = 10)	PPID (n = 10)
Total cortisol metabolites	41.0 ± 3.4	150.1 ± 34.0*^a^*
Cortisol	1.8 ± 0.2	7.5 ± 2.7*^a^*
Cortisone	0.6 ± 0.1	2.5 ± 0.9*^a^*
5*α*-THF	0.6 ± 0.1	2.0 ± 0.3*^a^*
5*β*-THF	0.4 ± 0.1	0.9 ± 0.2
5*β*-THE	0.2 ± 0.1	0.6 ± 0.1*^a^*
*α*-Cortol	0.6 ± 0.2	1.9 ± 0.6*^a^*
*β*-Cortol	5.7 ± 0.9	26.1 ± 7.0*^a^*
*α*-Cortolone	0.3 ± 0.04	1.3 ± 0.4*^a^*
*β*-Cortolone	4.6 ± 0.6	22.0 ± 7.7*^a^*
6*β*-Hydroxycortisol	1.3 ± 0.2	3.2 ± 0.7*^a^*
20*α*-Dihydrocortisol	1.2 ± 0.2	5.8 ± 1.4*^a^*
20*β*-DHF	24.0 ± 2.6	110.0 ± 47.0*^a^*
Excretion ratios		
(5*α*-THF + 5*β*-THF)/5*β*-THE	5.0 ± 1.1	4.8 ± 0.4
Cortisol/cortisone	3.1 ± 0.8	3.0 ± 1.2
5*β*-THF/cortisol	0.2 ± 0.1	0.1 ± 0.2
5*β*-THE/cortisone	0.3 ± 0.1	0.4 ± 0.3
5*α*-THF/cortisol	0.3 ± 0.2	0.4 ± 0.2

Data are mean ± SD and expressed as ratio of cortisol and its metabolites to creatinine (μg/mmol). Total cortisol metabolites were calculated as the sum of 5*β*-THF + 5*α*-THF + 5*β*-THE + *α*-cortol + *β*-cortol + *α*-cortolone + *β*-cortolone + 6*β*-hydroxycortisol + 20*α*-dihydrocortisol + 20*β*-DHF. Ratios reflecting overall 11*β*-HSD1/2 activity (tetrahydrocortisols/tetrahydrocortisone), 11*β*-HSD2 activity (cortisol/cortisone), 5*β*-reduction of cortisol (5*β*-tetrahydrocortisol/cortisol) and cortisone (tetrahydrocortisone/cortisone), and 5*α*-reduction of cortisol (5*α*-THF/cortisol) were not different between the groups.

Abbreviations: 5*α*-THF, 5*α*-tetrahydrocortisol; 5*β*-THE, 5*β*-tetrahydrocortisone; 5*β*-THF, 5*β*-tetrahydrocortisol.

^a^ Following a Kolmogorov-Smirnov test for normality, comparisons between groups were by Mann-Whitney *U* tests, *P* < 0.05.

There was no difference in excretion of androgen metabolites between castrated males and females in either group, so data from both sexes were analyzed together. Total androgen excretion was increased fivefold in horses with PPID (Table 4). This difference was accounted for largely by increased excretion of testosterone and androstenediol.

**Table 4.** Urinary Androgen Metabolite Excretion in Healthy Horses and Horses With PPID

Characteristic	Healthy (n = 10)	PPID (n = 10)
Total androgen metabolites	1.63 ± 1.46	8.87 ± 6.46*^a^*
Testosterone	0.81 ± 0.70	3.64 ± 1.42*^a^*
Aetiocholanolone	0.23 ± 0.17	0.46 ± 0.17
Androsterone	0.07 ± 0.18	0.16 ± 0.24
Epiandrosterone	0.10 ± 0.20	0.53 ± 0.50
Dihydrotestosterone	Below LOD	0.76 ± 1.33
DHEA	Below LOD	Below LOD
Androstenediol	0.42 ± 1.08	2.94 ± 4.52*^a^*
3*α* 5*α* Tetra-hydrotestosterone	Below LOD	0.39 ± 0.88

Data are mean ± SD and expressed as ratio of cortisol metabolite to creatinine (μg/mmol). Total androgen metabolites were calculated as the sum of aetiocholanolone, androsterone, epiandrosterone, dihydrotestosterone, DHEA, androstenediol, and 3*α* 5*α* tetra-hydrotestosterone.

Abbreviation: LOD, limit of detection.

^a^ Following a Kolmogorov-Smirnov test for normality, comparisons between groups were by Mann Whitney *U* tests, *P* < 0.05.

### Measurements in adipose tissue and liver

**Figure 2.**
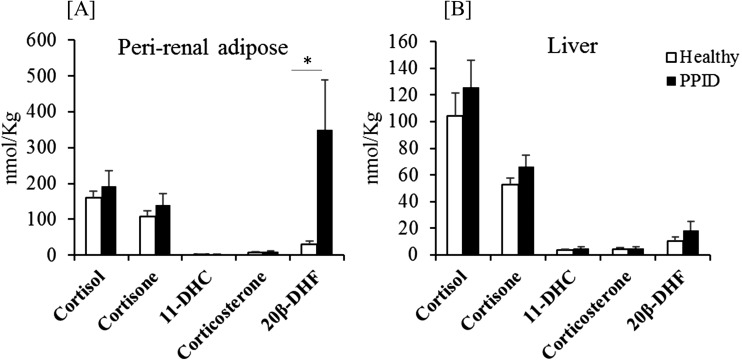
Tissue glucocorticoid concentrations in healthy horses and horses with PPID. (A) Perirenal adipose 20*β*-DHF concentrations were significantly higher in horses with PPID compared with healthy horses. (B) Individual steroid concentrations in liver did not differ between healthy and PPID horses. Data are mean ± SEM. *P < 0.05. 11-DHC, 11-deoxycorticosterone.

Horses with PPID had higher concentrations of 20*β*-DHF in the perirenal adipose (Fig. 2A). Cortisol concentrations were not different in the adipose or liver between the groups (Fig. 2A and 2B). Adipose to plasma ratios of cortisol did not differ significantly between the groups (healthy 1.8 ± 0.4 vs PPID 2.1 ± 0.5).

**Figure 3.**
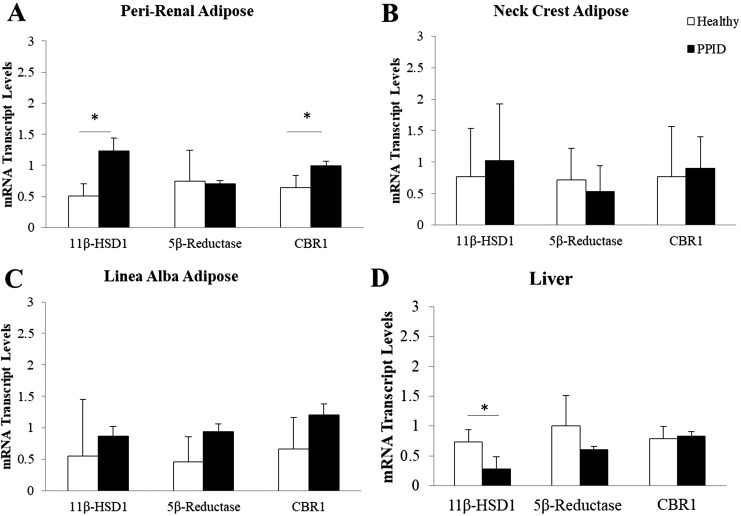
mRNA transcript levels of glucocorticoid metabolizing enzymes in three adipose depots and liver of healthy horses and horses with PPID. (A) 11*β*-HSD1 and CBR1 mRNA transcript levels were increased in perirenal adipose of horses with PPID; (B) neck crest and (C) linea alba adipose transcript levels were not different between the groups. 5*α*-Reductase was not identified in adipose tissue of the horse. (D) Hepatic 11*β*-HSD1 transcript levels were decreased in horses with PPID. Data are mean ± SEM. *P < 0.05.

Transcript levels of 11*β*-HSD1 were higher in perirenal adipose tissue and lower in liver of horses with PPID compared with healthy horses but not different in other adipose depots (Fig. 3A–3D). In contrast to other species (25), 5*α*-reductase transcripts were not detected in equine adipose, but 5*β*-reductase transcripts were. Transcript levels of 5*β*-reductase, which metabolizes cortisol to cortols and cortolones, were not different in adipose tissue between the groups. Transcripts of CBR1, the enzyme which converts cortisol to 20*β*-DHF (11), were more abundant in perirenal adipose tissue in PPID horses but not different in the other adipose depots or liver. There were no significant differences in GR expression between the groups in any of the adipose depots or the liver. Sex did not affect the patterns of expression of any of the genes analyzed.

### Measurements in pituitary tissue

**Figure 4.**
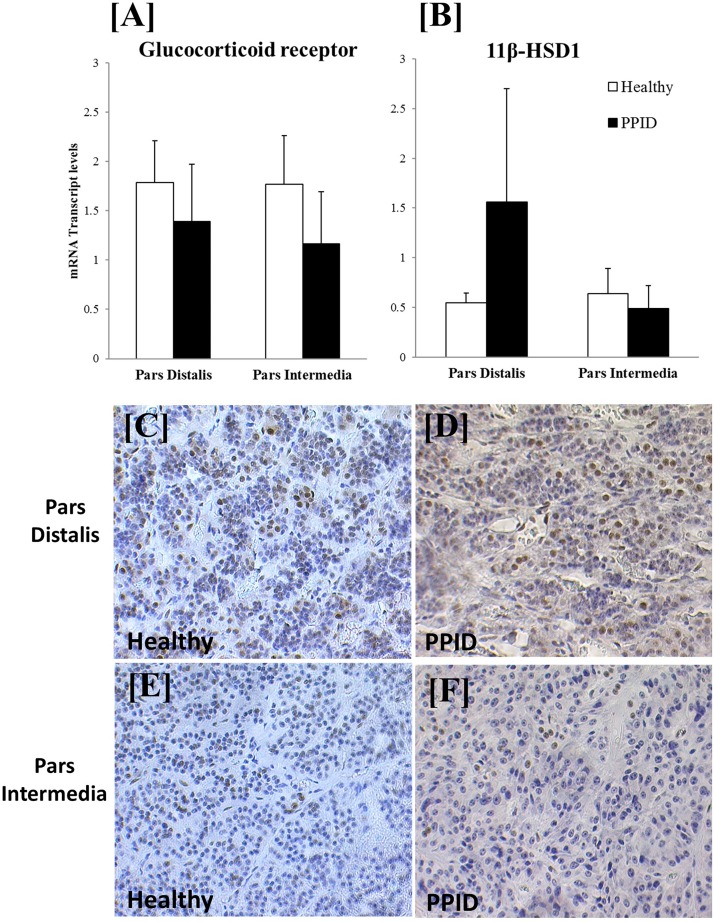
mRNA transcript levels of GR and glucocorticoid metabolizing enzymes in the pars distalis and pars intermedia of healthy horses and horses with PPID. mRNA transcript levels of (A) GR and (B) 11*β*-HSD1 were expressed in the pars distalis and pars intermedia, and levels did not differ between healthy horses and horses with PPID. (C–F) Photomicrographs of the pars distalis and pars intermedia of a (C, E) healthy horse and a (D, F) horse with PPID stained for GR (brown staining).

GR transcripts were identified in both the pars distalis and the pars intermedia of the equine pituitary, and there was no difference in expression between healthy horses and those with PPID (Fig. 4A); this was confirmed by immunohistochemistry (Fig. 4C–4F). 11*β*-HSD1 expression was not different between the groups (Fig. 4B).

## Discussion

We hypothesized that, despite apparently normal total plasma cortisol concentrations, PPID is a syndrome in which there is dysregulation of glucocorticoid metabolism or protein binding, resulting in enhanced tissue glucocorticoid action. Our data are, in part, consistent with this hypothesis; we demonstrated that horses with PPID have a fourfold increase in urinary excretion of glucocorticoid metabolites consistent with enhanced cortisol metabolism and clearance, as well as enhanced urinary androgen metabolite excretion consistent with adrenal activation by ACTH and increased clearance (26). In addition, we demonstrated decreased 11*β*-HSD1 expression in liver and increased 11*β*-HSD1 and CBR1 expression in perirenal adipose accompanied by increased 20*β*-DHF concentrations. We did not demonstrate, however, any differences in CBG binding or free fraction of cortisol, in contrast to one previous study that demonstrated an increased free cortisol fraction in horses with PPID (27). This discrepancy in findings may be due to the different methods used for measuring total glucocorticoids or reflect the diversity of clinical presentations encountered in PPID. There was a trend in our data for a lower cortisol binding capacity, and therefore, we may have been underpowered to detect more subtle differences. We did not measure salivary (28) or tear (29) cortisol concentrations, which are elevated in PPID and may be more sensitive indicators of the free cortisol available to tissues.

Urinary cortisol and “corticoids” have previously been measured in horses with and without PPID, and authors have concluded that this is unreliable as an indicator of disease due to the large overlap between groups (30, 31). We found striking differences in urinary steroids between groups, most likely because we measured a panel of urinary metabolites by highly sensitive LC-MS/MS. This method showed that the predominant glucocorticoid metabolite in horses is 20*β*-DHF (11), and as in other species, cortisol is a relatively minor metabolite in urine, so studies measuring cortisol by immunoassay are unlikely to have reliably detected differences between healthy horses and those with PPID.

Horses have a large capacity to increase urinary cortisol clearance; at exercise, clearance can increase by almost threefold, and the compensatory increase in cortisol secretion rate is reflected in an unchanged plasma cortisol (32). In obese horses, there is also a threefold increase in cortisol metabolite excretion without a change in plasma cortisol (11). Variation in cortisol clearance is emerging as an underrecognized independent regulator of HPA axis function, putatively contributing to compensatory HPA axis activation in metabolic syndrome, obesity (33–36), and underlying adrenal androgen excess in polycystic ovary syndrome (37). The fourfold increase in urinary metabolite excretion in horses with PPID, along with the increased androgen excretion, demonstrated in this study could be a compensatory response to increased cortisol (and androgen) secretion due to primary pathology in the pituitary and elevated plasma ACTH and *α*-MSH (38). Alternatively, it is plausible that there is a primary increase in cortisol clearance with chronic compensatory activation of the HPA axis driving the pathological changes in the pituitary. The normal plasma cortisol levels support the latter mechanism rather than the former. It is important to note that we have not measured clearance directly in this study, and further studies using labeled tracers (37, 39) or mathematical modeling of cortisol appearance and elimination (40, 41) would enable us to address this alternative hypothesis more thoroughly but are not feasible in the clinical environment.

We sought evidence of variations in cortisol metabolizing enzymes in liver and adipose tissue in horses with PPID because these might determine tissue cortisol levels and contribute to altered cortisol clearance. Reduced cortisol regeneration by 11*β*-HSD1 in liver is likely to contribute to enhanced cortisol clearance in horses with PPID, as in obese humans (42), but did not measurably reduce local cortisol concentrations. We also found evidence of depot-specific dysregulation of glucocorticoid metabolism in adipose tissue. Horses favor cortisol metabolism to 20*β*-DHF by the enzyme CBR1, which was increased in adipose tissue. Perirenal adipose expression of 11*β*-HSD1 was also increased in horses with PPID in this study, a finding replicated in perirenal adipose of humans with Cushing disease (43). The adipose cortisol pool in horses forms a significant component of total body cortisol. In humans, adipose glucocorticoid content is ∼27 nmol/kg (44), but we found that the glucocorticoid content of equine adipose was >100 nmol/kg. The ratio of adipose tissue cortisol to total plasma cortisol in humans is between 1:10 and 4:10 (45, 46), whereas in the horses in this study, it was close to 2:1. These data suggest that the adipose tissue in horses is a significant site of glucocorticoid storage and metabolism and that change in the size or enzymatic activity of the adipose depots could have a disproportionately large effect on whole-body glucocorticoid kinetics. Corticosterone concentrations in adipose were very low compared with plasma levels in both groups. This is expected given that equine adipose tissue expresses the transporter ABCC1, which exports corticosterone but not cortisol, but does not express ABCB1, which exports cortisol but not corticosterone (47).

Some authors have suggested that the diseased pars intermedia is unreceptive to negative feedback due to a lack of GR (15). Our data do not support this hypothesis as GR was present in the pars distalis and pars intermedia and not reduced in PPID cases. In humans, it has been suggested that adenomas of the anterior pituitary (pars distalis) are associated with an increase in 11*β*-HSD2 and a decrease in 11*β*-HSD1 expression that reduces cortisol in the pituitary and therefore reduces negative feedback (48). We did not find evidence of a similar phenomenon in horses because there were no significant differences in 11*β*-HSD1 or 11*β*-HSD2 expression in the pars distalis or the pars intermedia from horses with pituitary pathology.

It is possible that the pathology of PPID is exacerbated by androgen excess. Urinary androgens have not been measured in horses with PPID previously, but our findings suggest that hyperandrogenism occurs in parallel with HPA axis activation and might play a role in PPID as it does in human diseases characterized by HPA axis activation (such as polycystic ovary syndrome) (49).

This study is clinical and observational and as such has several limitations; the two groups differed significantly in age, which may confound the differences in glucocorticoid metabolism and transport. In humans, there is an age-related decrease in glucocorticoid excretion, especially in A-ring reduction, which is abolished in diseases such as Alzheimer’s disease in which the HPA axis is activated (50). Further work is required to determine the effect of age on cortisol metabolism and clearance in healthy horses. The sex distribution of the groups was also a limitation of the study, but the only differences detected between the sexes were in plasma testosterone. In mares, testosterone and androstenedione are secreted from both the adrenals and the ovaries, and both are susceptible to ACTH stimulation and stage of the mare’s cycle (51), which was not accounted for in this study, whereas the only source of androgens in the gelding are the adrenals. There were no differences in cortisol metabolite excretion, plasma glucocorticoid concentrations, or gene expression by sex.

In conclusion, we propose that the paradox of normal plasma cortisol concentrations in horses with PPID may be best explained by alterations in peripheral cortisol metabolism, which result in compensatory activation of the HPA axis together with altered local steroid concentrations within target tissues. Intriguingly, this raises the possibility that the pathognomonic changes of hyperplasia and nodularity in the pars intermedia, as well as the commonly reported hyperplasia of the pars distalis (15, 52), could be “secondary” or “tertiary” in the face of chronically enhanced cortisol clearance. The analogy is with hyperparathyroidism in renal failure in which secondary activation of parathyroid hormone secretion can progress to autonomous hyperplasia and adenoma formation (53). Reducing cortisol action through lowering ACTH remains a valid therapeutic approach but might be complemented, paradoxically, by new therapies that reverse the enhanced cortisol clearance.

## Abbreviations:

^3^H-cortisol: [1,2,6,7]-^3^H_4_ cortisol
11*β*-HSD1: 11*β*-hydroxysteroid dehydrogenase type 1
11*β*-HSD2: 11*β*-hydroxysteroid dehydrogenase type 2
20*β*-DHF: 20*β*-dihydrocortisol
CBG: corticosteroid binding globulin
CBR1: carbonyl reductase 1
GR: glucocorticoid receptor
HPA: hypothalamic-pituitary-adrenal
*HPRT*: hypoxanthine-guanine phosphoribosyltransferase
LC-MS/MS: liquid chromatography–tandem mass spectrometry
PPID: pituitary pars intermedia dysfunction
RT: room temperature
*SDHA*: succinate dehydrogenase A
*α*-MSH: *α*–melanocyte stimulating hormone
